# Deep Carious Lesions Management with Stepwise, Selective, or Non-Selective Removal in Permanent Dentition: A Systematic Review of Randomized Clinical Trials

**DOI:** 10.3390/healthcare11162338

**Published:** 2023-08-18

**Authors:** Nicola Figundio, Pedro Lopes, Tamara Kerber Tedesco, Juliana Campos Hasse Fernandes, Gustavo Vicentis Oliveira Fernandes, Anna Carolina Volpi Mello-Moura

**Affiliations:** 1 Faculty of Dental Medicine, Universidade Católica Portuguesa, 3504-505 Viseu, Portugal; 2 Centre for Interdisciplinary Research in Health (CIIS), Universidade Católica Portuguesa, 3504-505 Viseu, Portugal; gustfernandes@gmail.com; 3 Orthodontics and Pediatric Dentistry, Dental School, University of Sao Paulo, São Paulo 05508-000, SP, Brazil; 4 Periodontics and Oral Medicine Department, University of Michigan School of Dentistry, Ann Arbor, MI 48109, USA

**Keywords:** selective removal, total removal, deep carious lesion, dental restoration, pulpal exposure, systematic review, permanent teeth

## Abstract

Objective: The goal of this systematic study was to investigate the effectiveness of selective, stepwise, and non-selective removal techniques for caries removal in permanent teeth with deep carious lesions. The primary focus was the results found comparing techniques for caries removal to check whether there was pulp exposition; the secondary was the materials used for pulp protection and clinical findings reported within the included studies. Methods: The search was performed in two databases (PubMed/MEDLINE and Web Of Science). The studies included in this systematic review were selected based on eligibility criteria. The inclusion criteria were: (1) randomized controlled trials (RCTs), (2) that compared the total removal of carious tissue with selective removal in permanent teeth with deep carious lesions, (3) with a follow-up period of at least 6 months, and (4) publications in English. Regarding the exclusion criteria, the following were not considered: (1) articles published in other languages, (2) articles that did not compare the different types of total/selective decay removal, and (3) articles published before January 2008. The risk of bias and the quality of the included studies were independently assessed by two reviewers using the RoB 2 tool. Results: 5 out of 105 potentially eligible studies were included. Regarding the teeth included in the study, three articles performed management only on permanent molars, while other studies also performed management on incisors/canines/premolars/molars. Management protocols were divided into nonselective caries removal and partial caries removal (selective/stepwise). The theory of non-selective caries removal was considered an excessive, unnecessarily invasive option and a form of outdated management, and selective removal was preferred. Conclusion: The selective removal technique presented a higher success rate and fewer incidences of pulpal exposure than total removal, after up to 18 months of follow up. Moreover, only one session seemed to be a better management choice compared to two sessions because the cavity re-opening procedure is more prone to pulp exposure and highly depends on patient commitment. Otherwise, at 5 years of follow up, there was no difference between selective removal and total removal in management longevity. In addition, there were also no differences between the success of the materials used for definitive restorations in teeth subjected to any of the techniques evaluated.

## 1. Introduction

Dental caries is a widespread public health problem and the most common oral pathology worldwide. If left untreated, it is a disease capable of negatively impacting the quality of life and productivity of those affected [1]. Dental caries affect 2.5 billion adults and 573 million children, constituting a significant burden to healthcare systems and society [2,3]. Caries is a dynamic process, and it is important to understand its clinical manifestation [4]. Before the carious lesion becomes deep, it goes through different stages of progression [5]. When the enamel demineralization process (DES-RE reaction) is in its initial phase, accompanied by rapid loss of calcium and phosphorus ions, an incipient or enamel lesion can be found and is considered active. On the other hand, if the demineralization process stops, resulting from the DES-RE chemical reaction, which reverses the direction, it is now an inactive enamel carious lesion [6].

Necrotic and contaminated areas can be identified histologically, and their removal is recommended. However, if a firm and hard dentin is present in the cavity, it can be preserved. As the bacterial biofilm intensity increases, concomitantly, the inflammatory response intensifies [5]. If the carious invasion is not removed, the pulpal inflammation progresses, and the dentin will be exposed to bacterial invasion. This will lead to further demineralization and eventual deep cavitation, affecting the pulp. This will then result in irreversible pulpitis and pulpal necrosis [7].

One of the treatments for treating deep carious lesions is nonselective caries removal. This technique requires the removal of all demineralized and bacteria-contaminated dentin [8]. Otherwise, another concept advocates for selective removal; therefore, there is no consensus on the volume of decayed tissue that needs to be removed before restoration [8]. Evidence favoring selective caries removal suggests the advantages of preserving tooth vitality, avoiding pulpal damage, and, consequently, needing to be referred for endodontic treatment or even extraction [1]. The tooth must be restored with a material that promotes a good seal, preventing bacterial growth from affecting the tooth that remains in the decayed tissue. This type of management is considered successful when the tooth remains symptom-free, functional, and with a healthy pulp [9].

However, knowledge of evidence-based science related to the management possibilities involving deep carious lesions in permanent teeth is important to guide and encourage clinicians in making this management decision. Thus, the goal of this systematic study was to investigate the effectiveness of the selective, stepwise, and non-selective removal techniques for caries removal in permanent teeth with deep carious lesions. The primary focus was the results found comparing techniques for caries removal to check whether there was pulp exposition, observing the success rates; the secondary was the materials used for pulp protection and the clinical findings reported within the included studies. The null hypotheses were: (1) there will be similar results for all techniques; (2) similar results will be found for all of the protective materials used.

## 2. Materials and Methods

### 2.1. Study Design, Sources, and Search Strategy

The following systematic review was developed based on the PRISMA guidelines [10] and it was not registered. A bibliographic search was performed using PubMed/MEDLINE and Web of Science databases to collect articles published in the last 15 years (between January 2008 and January 2023), which is an adequate interval to find possible evolution in caries management. One limitation was the inclusion of articles only in the English language. The PICO strategy [11] (P = patients; I = intervention; C = comparison; O = outcomes) was used with the following question: “In permanent teeth with deep lesions (P), which of the following management (selective or stepwise removal of decayed tissue [I] or total removal [C]) would be the best option to reach the best result and the clinical success (O)?” The search strategy was specifically developed for each database: (1) PubMed/MEDLINE: ((“permanent teeth” OR “permanent tooth” OR “permanent dentition” AND “deep caries” OR “stepwise”) AND (“partial caries removal” OR “stepwise caries removal” OR “pulp vitality” OR “healing rate”)); (2) Web Of Science: ((“permanent teeth” OR “permanent tooth” OR “permanent dentition” AND “deep caries” OR “stepwise”) AND (“partial caries removal” OR “stepwise caries removal” OR “pulp vitality” OR “healing rate”)). Two reviewers (N.F. and A.C.V.M.N.) independently performed the entire screening and article selection process, as well as data collection and risk of bias assessment. A third researcher (T.K.T.) with experience treating deep carious lesions resolved conflict or doubt in the case of divided opinions.

### 2.2. Eligibility Criteria

Studies included in this systematic review were selected based on eligibility criteria. Inclusion criteria were: (1) randomized controlled trials (RCTs), (2) comparing total removal of carious tissue with selective or stepwise removal in permanent teeth with deep carious lesions, (3) with a follow-up period of at least 6 months, and (4) publications in English. Regarding the exclusion criteria, the following were not considered: (1) articles published in other languages, (2) articles that did not compare the different types of total/selective decay removal, and (3) articles published before January 2008.

### 2.3. Selection of Studies and Data Extraction

Two independent researchers (N.F. and A.C.V.M.M.) filtered the articles included in the study, analyzing the title and abstract for the study selection. Any disagreements between reviewers were discussed with a third author (T.K.T.). This study used Rayyan’s Intelligent Systematic Review Platform [12] to assist in the systematic review article selection process.

Reviewers independently extracted data from articles selected for analysis. A thorough analysis of the data was performed. The information collected during data collection were: title, year of the publication, authors, Ethics Committee registration, registration, and protocol location (if there was one or not), guideline, study design, sample size, groups, inclusion and exclusion criteria, type of material and management, outcome, follow up, type of statistical analysis, the tool used for data analysis, conclusions, and conflicts of interest.

### 2.4. Quality Assessment of the Included Studies

The risk of bias and the quality of the included studies were independently assessed by two reviewers (N.F. and A.C.V.M.M.) using the RoB 2 tool (Cochrane Risk of Bias Tool for Randomized Trials) [13]. This tool is structured in five domains (D) where the bias can be evaluated. Study quality was assessed in three categories: high risk of bias, few concerns, and low risk of bias. The quality assessment aimed to estimate the relative effect of two interventions or intervention strategies proposed in a clinical study and which produced the result. The ratings obtained were verified by a third reviewer (T.K.T.).

## 3. Results

### 3.1. Selection of Studies

**Figure 1:**
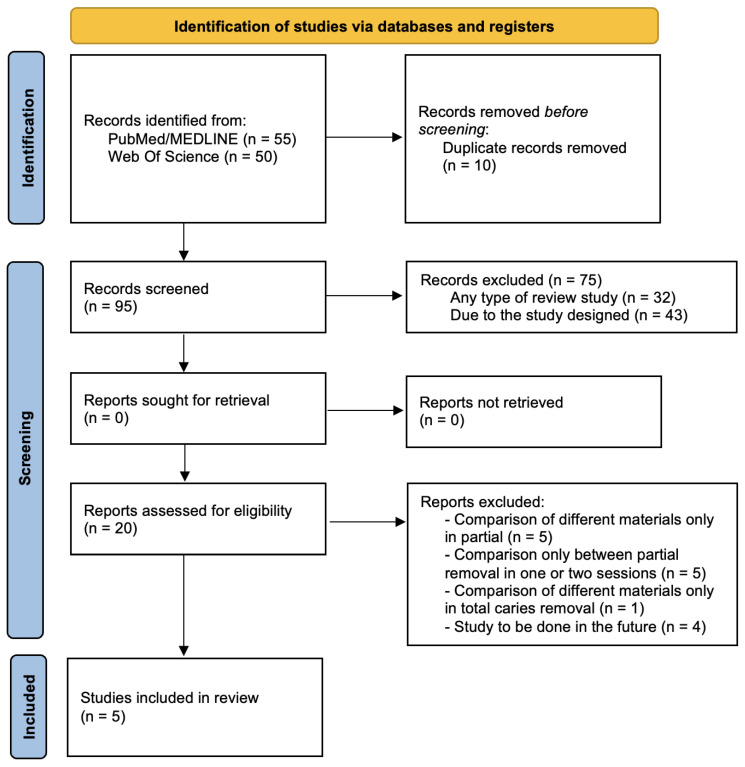
Flowchart for the studies’ selection.

Initially, 105 articles were identified in electronic databases (PubMed [*n* = 55]; Web Of Science [*n* = 50]). Of the 105 articles found through the search strategy, 10 duplicate articles were removed. A total of 95 articles remained and were reviewed by title and abstract. By checking by title and abstract, 75 articles that did not meet the inclusion criteria were excluded (k = 0.92) (reasons: review studies and design of the project/study). After applying the inclusion and exclusion criteria, the remaining 20 articles were considered for the full-text reading, which led to the exclusion of 15 articles. The reasons for these exclusions were: 5 compared different materials in only partial removal; 5 compared only partial removal in one or two sessions; 1 compared different materials only in nonselective caries removal; and 4 had an absence of numerical data. Therefore, the remaining 5 articles met the eligibility criteria and were included in this study (Table 1). The designs presented were: 5 randomized clinical trials [14,15,16,17,18] developed between 2010 and 2021 (k = 1). The flowchart diagram with the study selection process is shown in Figure 1.

### 3.2. Characteristics of the Studies

Table 2, Table 3 and Table 4 show the characteristics of the included studies. The Ethics Committee approved all articles [14,15,16,17,18] that were selected before starting and registered for data protection. One was a single study [14], whereas the others presented guidelines for the study design. The articles evaluated and proposed excavation techniques to treat deep carious lesions to guarantee greater longevity. Regarding the inclusion and exclusion criteria from the studies, similar clinical parameters were found in order to select and include patients. As for the teeth included in the study, three studies [15,16,18] performed management only on permanent molars, while other studies [14,17] also performed management on incisors/canines/premolars/molars. Management protocols were divided into nonselective caries removal and partial caries removal (selective/stepwise).

The materials were selected and used in all included studies considering whether they were total or partial caries removal. Except for two clinical studies [14,15], the remaining studies accurately referred to information about the materials applied, which were: liner (calcium hydroxide, Dycal^®^), provisional restoration (glass ionomer cement, Ketac Molar^®^), and definitive restoration (Herculite^®^ Tetric N-Ceram resin; Ivoclar Vivadent or amalgam). The clinical follow ups were considered in terms of short-term, from 1 to 18 months [14,17,18], and long-term (5 years) [15,16].

The challenges reported in the articles were pulp vitality, absence of periapical lesions based on thermal-electric tests, radiographic analysis, and lack of symptoms as tools for clinical success/failure. During the follow ups, the management was evaluated by external examiners; otherwise, two studies [14,15] did not describe it clearly. Several tools for statistical analysis were used and described in Table 3.

When comparing the nonselective caries removal technique with the selective and stepwise removal procedures for decayed dental tissue, Bjørndal et al. [16,18], Khokhar et al. [17], and Oz et al. [15] proposed that there were statistically significant differences in terms of longevity, marginal integrity, and success rate of the restorations. In contrast, according to their study, Ahmed et al. [14] guaranteed the inexistence of statistically relevant divergences between procedures.

### 3.3. Methodological Quality Assessment

**Figure 2:**
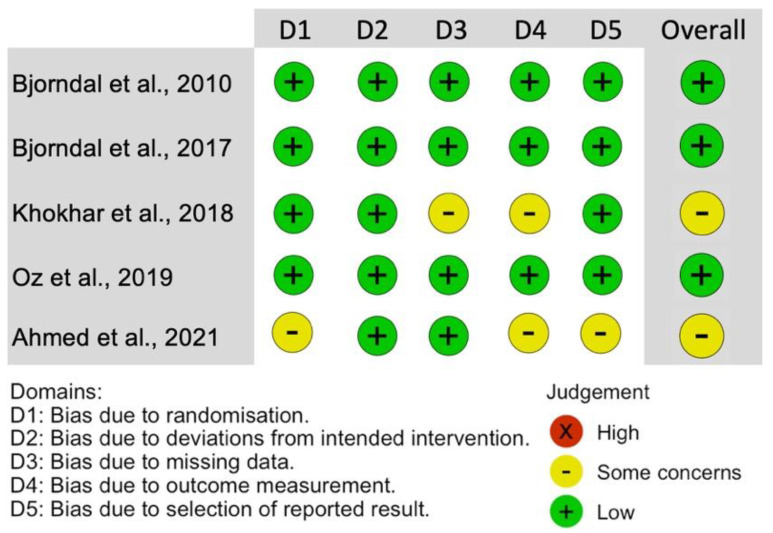
Quality assessment and risk of bias for the included studies [14,15,16,17,18].

Figure 2 presents the criteria applied to analyze the methodological quality of the studies using the RoB2 tool, as well as their respective responses. It is possible to observe, in general, that most of the criteria analyzed supported “low-risk” responses in all included studies [16,17,18], except for two that achieved some concerns [14,15]. Oz et al. [15] was the only study that presented an imbalance in the distribution of the two intervention protocols, in addition to not being clear on the choice of the definitive restorative materials (amalgam/composite resin) and the presence of external examiners; this study was thus classified as of “some concerns”. Ahmed et al. [14] did not describe whether a blinded assessment was performed or who the evaluators were and did not present a description of the possible sample loss in the follow-up (dropout).

## 4. Discussion

In the presence of a deep carious lesion, it is mandatory to know the possibilities of procedures that can be followed for caries removal. The decision on which procedure to follow should be made based on technical analysis, looking for the most favorable result, i.e., dental tissue preservation, pulp vitality maintenance, and marginal integrity preservation for future restoration. Even though nonselective caries removal is still popular among dentists, it is not highly recommended for deep lesions. It may be an unnecessary form of management [1], often resulting in pulpal exposure.

Total carious tissue removal consists of removing all the carious dentin (infected and affected) from the surrounding walls and the bottom of the cavity, since its main purpose is to eliminate bacteria and all the caries-infected and affected biomass [1,19]. On the other hand, gradual removal (SWR) is a two-stage technique for removing carious lesions, with the first stage removing caries from the soft dentin and placing a temporary restoration to seal; the second stage is removing the temporary restoration (re-entry), removing caries from the solid dentin, and placing a permanent restoration [2]. While the selective removal technique (PCR) corresponds to partial removal of carious dentin in the back walls, total removal in the surrounding walls and the tooth is restored in a single phase [20].

Thus, the present systematic review included only RCTs that responded to the following question: “In permanent teeth with deep lesions, which of the following management (selective or stepwise removal of decayed tissue or total removal) would be the best option to reach the best result and the clinical success?”.

### 4.1. Caries Removal

The literature shows a higher success rate for the selective removal technique compared to stepwise removal, suggesting no need for re-entry 3–5, maintaining pulpal health. Therefore, both are more conservative approaches. In the non-selective technique, even though it is a non-conservative method, it is recommended in non-deep carious lesions. Our meta-analysis, despite the heterogeneity present caused by the diversity of analysis of the articles included, showed that the selective removal technique tends to be the best management option in the short term (1.5 years follow up); otherwise, at five years, the results were similar, permitting the choice of any technique. Therefore, the selective removal of decayed tissue has the benefit of reducing the risk of pulpal exposure. Moreover, this study compared the difference in the risk of selective removal of decayed tissue in permanent teeth with gradual selective and non-selective removal. Similar to Barros et al. [5], our meta-analysis showed a statistically significant difference in favor of the selective removal of decayed tissue in the short term. This analysis agrees with Li et al. [21], another systematic review showing a substantial reduction in the risk of pulpal exposure using selective caries removal compared to non-selective caries removal.

As a single-session removal procedure, all studies [14,15,16,17,18] performed a common technique, removal of carious tissue from the lateral walls and the enamel-dentin junction, using low-speed carbide burs and/or a manual excavator. The removal limits were also uniform, removing superficial necrotic dentin from the pulpal and axial wall using a low-speed round drill. A layer of soft and moist carious dentin was left adjacent to the pulpal wall. In a clinical study found, Banerjee et al. [22] stated in detail that the dentin located on the pulp wall should be coriaceous, while the cavity margins and peripheral dentin should be free of carious tissue, with hardened dentin. As explained by Bjørndal et al. [23], in the case of the gradual removal of carious tissue, the cavity is re-opened after 8–12 weeks, and the final excavation is conducted, leaving only yellowish or grayish central hard dentin.

Additionally, three included studies [15,16,17] used distilled water for washing the cavity at the end of removal and caries detectors in cases of non-selective removal in order to remove all carious tissue. Khokhar et al. [17], authors of an included study, stated that the dye reduces visual and tactile subjectivity. However, it is less specific for caries, resulting in excessive removal of totally healthy tooth structure and a greater likelihood of mechanical pulp exposure.

Hoefler et al. [24], through a systematic review, argued in favor of selective caries removal, stating that the technique of gradual removal, compared to the selective removal of decayed tissue, always requires at least two visits/interventions. Moreover, the possibility of pulpal exposure is greater during the re-opening procedure of the cavity. Furthermore, a larger number of materials are used and, therefore, the cost of the management can be higher. It is important to have adequate marginal adhesion when applying temporary and permanent restorations to avoid leakage and protect the remaining tooth tissue from bacteria. Infected dentin or carious lesions negatively influence the adhesion of bonding agents compared to healthy dentin, reinforcing the importance of the surrounding cavity walls and enamel being completely free of carious tissue [19]. The softer layer, demineralized dentin left during selective excavation of the carious tissue, has a lower bond strength to the adhesive and may not withstand the functional load [1].

### 4.2. Type of Management Age-Related

Regarding the age of the patients, Bjørdnal et al. [23] stated that it did not influence the management outcome without exposure but that the failure rate is related to patients with mild symptoms detailed, not described, and severe caries. In agreement with this, Maltz et al. [25] described no consensus on the importance of management success or failure concerning age. Khokhar et al. [17] clarified they included teeth with >50% caries depth, unlike other studies, and Bjørndal et al.’s study [16], which included teeth with >75% caries depth in the non-selective removal group, found that total removal in their study caused less pulpal exposure. Therefore, the authors stated that total removal is no more effective than selective removal in permanent teeth.

### 4.3. Base Material and Techniques of Restoration

Differences that emerged in the included studies concerned the base material to be placed after the respective removal techniques. Bjørndal et al. [16,18] and Oz et al. [15] used calcium hydroxide as the base (Dycal; DeTrey Dentsply, Skarpnäck, Sweden), while Khokhar et al. [17] and Ahmed et al. [14] used resin-modified glass ionomer (GC; Fuji Lining LC, Tokyo, Japan). Khokhar et al. [17] stated that calcium hydroxide was not used due to high solubility and hydrolysis time, which gives the adhesion areas low compressive strength and a lack of adhesion to the tooth. A study in the literature found that calcium hydroxide liners should only be used in the deepest points of the cavity, where the remaining dentin thickness is ≤0.5 mm. A protective layer of resin-modified glass ionomer should always follow the application of calcium hydroxide liners [1]. However, recent studies indicate that it is not yet possible to make a conclusive statement about the superiority of either type of luting material as a cavity base [2]. A resin-modified glass ionomer cement may be advised. However, they need further high-quality, long-term randomized control studies [2].

Regarding the techniques of provisional restoration after the gradual removal and placement of the base, Bjørndal et al. [23] stated that when using a zinc oxide–eugenol cement, the possibility of failure is higher, favoring a glass ionomer restorative cement. In response, in another piece of evidence, Maltz et al. [25] stated that regardless of the restorative material, whether zinc oxide–eugenol, MRI, or glass ionomer restorative cement, all studies of gradual removal had the risk of patients dropping out after 6 to 12 months for the second visit, which may indicate a low success rate. Therefore, choosing an appropriate technique for sealing the cavity is more important than selecting the type of restorative material. Also, Maltz et al. [25] asserted that the type of restorative material may also influence the longevity of the restoration, as well as clinical aspects and patient- and practitioner-related characteristics.

### 4.4. Final Considerations

Carious lesions can be treated conservatively, mainly by controlling the etiological factors of the carious process. This strategy includes dietary modification, biofilm rupture, and the hermetic sealing of the carious biofilm from its nutrient supply [1]. Thus, from a practical point of view, the selective removal of carious tissue is effective without completely eradicating the entire bacterial population to maintain pulp health. It also decreases the risk of pulpal exposure.

All the articles included in this systematic review agreed and supported the theory that, according to the latest scientific evidence, the non-selective removal of caries is considered an excessive, unnecessarily invasive, and outdated form of management [1]. Therefore, according to the systematic review by Barros et al. [2], the technique of the selective removal of carious tissue may be used to treat deep caries to avoid pulpal exposure and preserve tooth structure without compromising the longevity of the restoration. However, there is a lack of studies on the need to perform the management in a single or double session [14,15,16,17,18].

## 5. Limitations

One of the most evident limitations of the current systematic review is the lack of scientific studies comparing the subjects used and the long-term clinical and radiographic results in permanent teeth between selective removal or gradual (stepwise) removal, i.e., in one or two sessions, versus total removal. Moreover, additional points must be observed among the studies, such as the variables involved in removing caries (turbine, micromotor, manual spoon, and professional experience); these factors may cause differences in the results and, in a systematic review, not including these factors is a limitation. We believe that these facts may interfere with the results found. RCTs are always important to increase the level of scientific evidence on a given subject; however, a limited number of studies were included and were available, so future studies could be extended to include other study designs. Questions remain about this theme, but they will certainly be answered, and the findings consolidated, by further studies.

## 6. Conclusions

Considering the limitations of this study, the selective removal technique presented a higher success rate and fewer incidences of pulpal exposure than total removal, after up to 18 months of follow up, rejecting the null hypothesis. Selective removal in one session seemed to be a good management option. The two-session selective removal technique (gradual removal) is not highly recommended because the cavity re-opening procedure is more prone to causing pulp exposure and highly depends on patient commitment. Otherwise, at 5 years of follow up, the null hypothesis was accepted. There was no difference between selective removal and total removal in terms of the management forms’ longevity. In addition, there were also no differences between the success of the materials used for definitive restorations in teeth subjected to any of the techniques evaluated. Furthermore, there is no conclusion about the best material to use as a liner/base at the bottom of the cavity and/or as a restorative. Further investigations with well-standardized methodology and statistical analysis, with a detailed comparison between materials and longer follow up, are needed to increase the level of scientific evidence.

## Figures and Tables

**Table 1:** Information on the articles included.

Article	Title	Year	Authors	Follow Up	Clinical Question	Ethics Register	Guideline for Drawing	Study Design Type	Exclusion of Duplicates
1	Treatment of deep caries lesions in adults: randomized clinical trials comparing stepwise vs. direct complete excavation, and direct pulp capping vs. partial pulpotomy	2010	Bjørndal et al. [17]	1 year	The two randomized clinical trials presented here were designed to test the effect of: (i) stepwise excavation vs. direct complete excavation of deep carious lesions in adults	Yes	Yes	Randomized clinical trial	yes
2	Randomized Clinical Trials on Deep Carious Lesions: 5-Year Follow-up	2017	Bjørndal et al. [16]	5 years	The aim of this article was to report the 5-year outcome on these previously treated patients presenting with radiographically well-defined carious lesions	Yes	Yes	Randomized clinical trial	yes
3	Outcomes of Partial and Complete Caries Excavation in Permanent Teeth: A 18 Month Clinical Study	2018	Khokhar et al. [17]	18 months	The aim of this study was to compare the clinical and radiographic outcomes of partial and complete caries removal (CCR) in permanent teeth with deep carious lesions	Yes	Yes	Randomized clinical trial	yes
4	Long-Term Survival of Different Deep Dentin Caries Treatments: A 5-Year Clinical Study	2019	Oz et al. [15]	5 years	The aim of this in vivo study was to evaluate the long-term clinical survival of different deep dentin caries treatment options	Yes	Yes	Randomized clinical trial	yes
5	Comparison of Partial and Complete Caries Excavation in Permanent Teeth: An 18 month Follow-up	2021	Ahmed et al. [14]	18 months	The aim of this study was to compare performance and survival of composite restorations in permanent teeth using partial caries removal (PCR) versus complete caries removal (CCR)	Yes	Yes	Randomized clinical trial	yes

**Table 2:** Materials and results analyzed in each of the articles included.

Authors/Year	Sample Size	Study Group	Type of Material	Outcomes
Bjørndal et al., 2010 [18]	314	Stepwise excavation vs. Direct complete excavation	Temporary material: calcium hydroxide (Dycal^®^). Temporary restorative: glass ionomer cement (Ketac Molar^®^) Final restoration: a resin material (Herculite^®^)	Stepwise excavation OR immediate complete removal Pulp vitality and absence of periapical lesions
Bjørndal et al., 2017 [16]	314	Stepwise excavation vs. Direct complete excavation	Temporary material: calcium hydroxide (Dycal^®^). Temporary restorative: glass ionomer cement (Ketac Molar^®^) Final restoration: resin material (Herculite^®^)	Stepwise excavation OR immediate complete removal Pulp vitality and absence of periapical lesions after 5 years
Khokhar et al., 2018 [17]	153	Partial caries removal vs. Direct complete excavation	Temporary restorative: resin-modified glass ionomer cement (RMGIC) Final restoration: composite resin (Tetric N-Ceram; Ivoclar Vivadent)	Partial caries removal OR immediate complete removal Pulp vitality and absence of periapical lesions
Oz et al., 2019 [15]	391	Stepwise excavation vs. Direct complete excavation	Temporary material: calcium hydroxide Temporary restorative: glass ionomer cement Final restoration: a resin material and amalgam	Stepwise excavation OR immediate complete removal Pulp vitality and absence of periapical lesions
Ahmed et al., 2021 [14]	70	Partial caries removal vs. Direct complete excavation	Temporary restorative: resin-modified glass ionomer cement (RMGIC) Final restoration: a resin material	Partial caries removal OR immediate complete removal Pulp vitality and absence of periapical lesions

vs. = versus.

**Table 3:** Statistical analysis and assessment tools of the included articles.

Authors/Year	Follow Up	Type of Statistical Analysis	Tool/Data Analysis
Bjørndal et al., 2010 [18]	1.5 y	Mann–Whitney U-test Chi-square test Binary logistic regression analysis	Pulp vitality » positive response to thermal (cold) or electrical stimulation Periapical radiolucency Unbearable pain Two blinded examiners
Bjørndal et al., 2017 [16]	5 y	χ^2^ test and Fisher’s exact test Kaplan–Meier plots and log-rank Cox regression analyses Intention-to-treat (ITT) analysis was performed (x^2^ test) Statistical significance at *p* = 0.05.	Pulp vitality » positive response to thermal (cold) or electrical stimulation Periapical radiolucency Unbearable pain Two blinded examiners
Khokhar et al., 2018 [17]	1, 3, 6, 12, and 18 months	Chi-square test. Statistical analyses SPSS version 20.0 software Statistical of significance at *p* ≤ 0.05	Pulp vitality » positive response to thermal (cold) or electrical stimulation Absence of signs and symptoms of irreversible pulpitis Periapical radiolucency Two blinded examiners
Oz et al., 2019 [15]	5 y	Kaplan–Meier Log-rank (Mantel–Cox) tests (α = 0.05)	Pulp vitality » positive response to thermal (cold) or electrical stimulation Absence of periapical lesions as well as a clinical symptom
Ahmed et al., 2021 [14]	6, 12, and 18 months	Chi-square test Analyzed in SPSS v23 software	Pulp vitality » positive response to thermal (cold) or electrical stimulation Absence of periapical lesions

y = year(s).

**Table 4:** Main results and conclusions of the included articles.

Authors/Year	Results	Conclusions
Bjørndal et al., 2010 [18]	Gradual excavation: fewer pulp exposures than direct full excavation (difference: 11.4%) 1-year follow up: success rate with graded excavation (difference: 11.7%) There were no significant differences in pulp vitality between the two capping procedures after more than 1 year (31.8% and 34.5%; difference: 2.7%)	Gradual excavation had a lower pulp exposure than direct full excavation SWR is most recommended for the management of deep carious lesions
Bjørndal et al., 2017 [16]	SWR had a higher success rate (60.2%) at a 5-year follow up than the non-selective carious removal (46.3%) (*p* = 0.031) The pulp exposure rate was lower in the SWR group (21.2% vs. 35.5%; *p* = 0.014) Regardless of pulp exposure status, the difference (13.3%) was still significant when considering sustained pulp viability without apical radiolucency and excruciating pain (95% confidence interval, 3.1–26.3, *p* = 0.045)	A SWR is most recommended for the management of deep carious lesions
Khokhar et al., 2018 [17]	Pulp exposure occurred in 13 (9.55%) cases of the CCR group A statistically significant difference (*p* = 0.001) in terms of pulp exposure was found between the two groups After 18 months, 123 teeth were evaluated (CCR = 56 and PCR = 67) and the success rate in the CCR group (98.21%) and the PCR group (92.53%) did not differ significantly (*p* = 0.115)	PCR is the elective treatment option for mature permanent teeth with deep carious lesions, even though similar success rate was found for CCR and PCR
Oz et al., 2019 [15]	Of a total of 214 patients evaluated, 126 received SWR management, 88 received CCR and 67 received DPC; the average of observation period was 62 months. The survival rates were 85.7%, 90.9%, and 59.7% for SWR, CCR, and DPC, respectively (*p* = 0.001).	SWR management preserves pulp vitality of deep dentin lesions instead CCR or DPC
Ahmed et al., 2021 [14]	In the CCR group, 25 patients had an occlusal lesion and 5 had an occlusal-proximal lesion. In the PCR group, 27 teeth were diagnosed with an occlusal lesion and 3 with an occlusal-proximal lesion At 18 months’ follow up, the success rate was 100% in the CCR group and 93.3% in the PCR group (*p* = 0.49) Pulp exposure occurred in 23.3% of procedures in the CCR group and none in the PCR group	PCR had similar success rates to CCR and is associated with a significantly smaller pulp exposure rate

CI = confidence interval; SWR = stepwise removal; CRC = complete caries removal; DPC = direct pulp capping; PCR = partial caries removal.

## Data Availability

The data used to generate and support the findings of this study are available within the study.
